# Community-driven roadmap for integrated disease maps

**DOI:** 10.1093/bib/bby024

**Published:** 2018-04-23

**Authors:** Marek Ostaszewski, Stephan Gebel, Inna Kuperstein, Alexander Mazein, Andrei Zinovyev, Ugur Dogrusoz, Jan Hasenauer, Ronan M T Fleming, Nicolas Le Novère, Piotr Gawron, Thomas Ligon, Anna Niarakis, David Nickerson, Daniel Weindl, Rudi Balling, Emmanuel Barillot, Charles Auffray, Reinhard Schneider

**Affiliations:** 1 Luxembourg Centre for Systems Biomedicine, Universite du Luxembourg, 7 Avenue des Hauts-Fourneaux, L-4362 Esch-sur-Alzette, Luxembourg; 2 Institut Curie, PSL Research University, INSERM U900, F-75005 Paris, France and CBIO-Centre for Computational Biology, MINES ParisTech, PSL Research University, F-75006 Paris, France; 3 European Institute for Systems Biology and Medicine, CIRI UMR5308, CNRS-ENS-UCBL-INSERM, Université de Lyon, 50 Avenue Tony Garnier, 69007 Lyon, France; 5 Computer Engineering Department, Faculty of Engineering, Bilkent University, Ankara 06800, Turkey; 6 Institute of Computational Biology, Helmholtz Zentrum München-German Research Center for Environmental Health, Ingolstädter Landstr. 1, 85764 Neuherberg, Germany; 7 Division of Systems Biomedicine and Pharmacology, Leiden Academic Centre for Drug Research, Faculty of Science, Leiden University, Leiden, Netherlands; 8 The Babraham Institute, Babraham Research Campus, Cambridge CB22 3AT, United Kingdom; 10 Faculty of Physics and Center for NanoScience (CeNS), Ludwig-Maximilians-Universität, 80539 München, Germany; 11 GenHotel EA3886, Univ Evry, Université Paris-Saclay, Evry 91025, France; 12 Auckland Bioengineering Institute, University of Auckland, Auckland, New Zealand

**Keywords:** disease maps, molecular biology, mathematical modeling, knowledge repository, biocuration, translational medicine, pathway representation

## Abstract

The Disease Maps Project builds on a network of scientific and clinical groups that exchange best practices, share information and develop systems biomedicine tools. The project aims for an integrated, highly curated and user-friendly platform for disease-related knowledge. The primary focus of disease maps is on interconnected signaling, metabolic and gene regulatory network pathways represented in standard formats. The involvement of domain experts ensures that the key disease hallmarks are covered and relevant, up-to-date knowledge is adequately represented. Expert-curated and computer readable, disease maps may serve as a compendium of knowledge, allow for data-supported hypothesis generation or serve as a scaffold for the generation of predictive mathematical models. This article summarizes the 2nd Disease Maps Community meeting, highlighting its important topics and outcomes. We outline milestones on the roadmap for the future development of disease maps, including creating and maintaining standardized disease maps; sharing parts of maps that encode common human disease mechanisms; providing technical solutions for complexity management of maps; and Web tools for in-depth exploration of such maps. A dedicated discussion was focused on mathematical modeling approaches, as one of the main goals of disease map development is the generation of mathematically interpretable representations to predict disease comorbidity or drug response and to suggest drug repositioning, altogether supporting clinical decisions.

## Introduction

The concept of disease maps emerged to bridge the domains of biological and computational research on various human disorders. In essence, these maps are representations of disease mechanisms that are both human and machine-readable [1–4]. Visual representation allows clinical and life sciences researchers to explore charted disease mechanisms, which are often complex and interconnected. Computer-tractable, standardized representation of the underlying information creates an interface to a broad range of bioinformatic workflows. As such, disease maps are an important platform with the potential to link the domains of biomedical knowledge and data, providing an intermediate step between a conceptual and an executable model.

In the recent years, the members of the Disease Maps Community (DMC) developed various disease maps resources, hand in hand with other groups around the globe. The community held its initial meeting in February 2017, hosted by the European Institute for Systems Biology and Medicine in Lyon, France. There we recognized a great potential in such type of exchange, especially because, despite different disease contexts, we face similar challenges, ranging from establishing proper tools and standards for knowledge encoding, through visualization of multidimensional data sets, to handling large and complex maps. We decided to meet regularly to help shape the direction where the project is heading. In October 2017, we held the 2nd DMC meeting, hosted by the Luxembourg Centre for Systems Biomedicine in Belval, Luxembourg. Here, we summarize this meeting, highlight important topics and outcomes of our discussions and propose a roadmap for the development of disease maps.

In this article, we first introduce the DMC and describe its rationale, mode of operation and spectrum of expertise. Next, we overview the 2nd DMC meeting, highlighting important topics and discussions of special focus. Then, we describe the milestones on the ‘Disease Maps Roadmap’, identified during a dedicated, extended discussion session during the meeting. In the last chapter of the article, we briefly summarize the outcomes and discuss further steps, including necessary standards and tools.

## The Disease Maps Community

The DMC (http://disease-maps.org/) is a group of developers and users of disease maps of various human disorders, including cancer, neurodegenerative and immune diseases. The community formed to exchange experiences and to establish best practices for creation, maintenance and application of disease maps. The group is composed of biomedical and clinical researchers with expertise on particular diseases [2, 3, 5], but also of bioinformaticians, computer scientists and mathematicians working on technologies supporting curation and exploration of the maps [6–8]. Because the community involves projects at different stages of development, upcoming disease maps can benefit from the experience of developers at the advanced stage. At the same time, new disease maps bring their own unique use cases providing new perspective for the adoption of curation standards and required technology developments. At the time of writing, researchers from France, Germany, Luxembourg, UK, Portugal, Spain and Turkey take part in the DMC. The participation in the community is voluntary.

**Figure 1:**
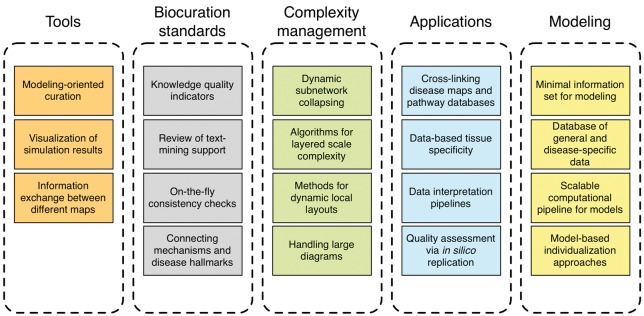
The milestones of the DMC roadmap. Five groups of topics are highlighted. Tools: Software and methods supporting the development and maintenance of the maps; Biocuration standards: standards for knowledge gathering and encoding in the maps; Complexity management: methods that handle inherent complexity and facilitate visual exploration of the contents of the maps; Applications: workflows where maps can be applied to support knowledge exploration, generation of new hypotheses or support clinical decisions; and Modeling: standards and tools allowing to refine the maps into executable mathematical models.

Regular meetings help to catalyze the exchange between the community members. The 1st DMC meeting allowed us to identify challenges shared across different disease maps' projects and recognize the value of exchanging best practices. Moreover, it was apparent that we need to keep track of our efforts to best align them. Therefore, the main objective of the 2nd DMC meeting (http://disease-maps.org/events) was to bring the community up to speed about the ongoing activities, introduce new members with their projects and engage into deep discussion on challenges, potential solutions and the next steps to take. This discussion was at the heart of our meeting, and is described in detail in the following section. Participants engaged in extensive discussions on critical topics for tools, applications, curation standards and complexity management. Moreover, an entire session was dedicated to the topic of mathematical modeling. Based on the outcome of our discussions, we outline the roadmap for disease maps development (Figure 1).

## Milestones on the ‘Disease Maps Roadmap’

The community discussed five aspects of the disease maps, namely: (i) tools supporting the development and use of the maps, (ii) standards needed for biocuration of the content, (iii) management of complex content, (iv) application of the maps in the biomedical domain and (v) the predictive modeling of disease mechanisms. We defined a number of milestones, summarized in Figure 1. Some of them span multiple aspects of disease maps. For instance, ‘encoding and use of models’ need to be solved at the levels of tools, biocuration and modeling methodology. Complexity management and tools share milestones for ‘dynamic network layout’, while biocuration and applications both define ‘quality indicators of encoded knowledge’ as a milestone.

### Tools for map creation, visualization and exploration

Disease maps are an emerging concept, bridging bioinformatics, molecular biology and clinical research. Appropriate tools are needed to support creation and use of the maps, including handling relevant standards for knowledge encoding, annotation and exploration. It is crucial to align new developments in this area with concrete use cases. In fact, the development of many available tools was initiated to directly address the needs of the DMC, and their further development takes into account the emerging challenges. Table 1 summarizes the tools discussed in the following text, both those already used for disease maps development and analysis, and those that offer new important functionalities.

**Table 1:** Summary of tools for creation and exploration of disease maps

Tool: description	Role	Web- oriented	Scale of maps	Data overlay	Supported standards	Active	Kinetics support	Used for disease maps
BiNoM^9^: Manipulating disease map diagrams, Cytoscape plugin	Explore Update	No	Large	Yes	BioPAX^10^ CellDesigner SBGN,^11^ SBML^12^	No	No	Yes
CellDesigner^13^: Construction of process diagrams and simulations for molecular biology	Construct	No	Large	No	CellDesigner	No	Yes	Yes
iPthways+^4^: Visualization of pathways and process diagrams	Explore	Yes	Large	No	CellDesigner	Yes	No	Yes
MINERVA^6^: Visualization and exploration of disease map diagrams	Explore	Yes	Large	Yes	CellDesigner SBGN	Yes	No	Yes
NaviCell^14^: Visualization and exploration of disease map diagrams	Explore	Yes	Large	Yes	CellDesigner	Yes	No	Yes
Newt^8^: Construction of pathways and process diagrams	Construct Explore	Yes	Medium	No	SBGN	Yes	No	Yes
PathVisio^15^: Construction of pathways and process diagrams	Construct Explore	No	Small	Yes	SBGN	Yes	No	Yes
Payao^16^: Visualization of pathways and process diagrams	Explore	Yes	Large	No	CellDesigner	No	No	Yes
SBGN-ED^17^ (VANTED): Construction of pathways and process diagrams	Construct	No	Medium	No	SBGN	Yes	No	Yes
yED^18^: Construction of pathways and process diagrams	Construct	No	Medium	No	SBGN	Yes	No	Yes
BioPAXViz^19^: Visualization of metabolic pathways	Explore	No	N/A	Yes	BioPAX	Yes	No	No
COBRA Toolbox^20^: Simulation and visualization of pathways	Explore	No	Medium	No	CellDesigner SBML	Yes	Yes	No
Escher^21^: Construction and simulation of metabolic pathways	Construct Explore	Yes	Medium	Yes	SBML SBGN	Yes	Yes	No
iVUN^22^: Visual analysis and simulation of kinetics in pathways	Explore	No	Small	Yes	SBML	No	Yes	No
NDex^23^: Sharing of network data for computational biology	Explore	Yes	N/A	No	Cytoscape^24^	Yes	No	No
Physiome Model Repository^25^: Sharing of cellular models	Explore	Yes	N/A	No	CellML^26^	Yes	Yes	No
SEEK^27^ (FAIRDOMHub): Sharing of SBML models and datasets	Explore	Yes	N/A	No	SBML	Yes	Yes	No

*Notes*: The table lists the tools that support construction and exploration of the disease maps' content, highlighting their role in the process. We indicate their capability to work over the Web (‘web-oriented’ column) and the size of the maps that they can handle (‘scale of maps’ column): large: over a thousand of elements, medium: hundreds of elements, small: under a hundred of elements. ‘data overlay’ column indicates which tools can overlay external data set on their content. ‘supported standards’ column lists which standard data formats are supported by a given tool. Even though ‘CellDesigner’ format is only a *de facto* standard, based on early versions of SBGN and SBML formats, we list it because of the popularity of the tool. Finally, we indicate, which of the tools are actively developed, support reaction kinetics and are currently used for disease maps' creation and exploration. SEEK and NDex platforms provide an automated layout of uploaded models, while BioPAXViz and Physiome model repository use layoutless formats (BioPAX and CellML), making the assessment of the scale imprecise.

#### Constructing maps

A key challenge in the field is the lack of tools tailored exactly to develop content for a disease map. Visual pathway editors [13, 17] that offer significant level of compatibility with Systems Biology Graphical Notation (SBGN) [11] are often used for this purpose, contributing to content reuse. Other solutions like Cytoscape-based Biological Network Manager (BiNoM) [9] or PathVisio [15] allow for importing, manipulating and exporting SBGN or CellDesigner formats. An interesting case is a graph editor yED [18] that introduced an SBGN palette, allowing drawing of graphs that look like SBGN diagrams.

Still, disease maps are frequently updated and extensively annotated knowledge repositories, and the mentioned editors have limited capabilities to support for such resources. Harmonization of curation standards (see section ‘Biocuration and knowledge representation standards’) is also difficult, as each of the mentioned tools uses its own encoding of the content, risking an inexact translation when transferring information between sources. An important development addressing this problem is the Web-based editor of diagrams encoded in SBGN: Newt [8]. The creators of Newt actively participate in the DMC, helping to shape and benefiting from the discussed roadmap. A milestone on the road toward mechanistic, modeling-oriented curation will be enabling support for the Systems Biology Markup Language (SBML) [12] (see section ‘Use of maps for mathematical modeling’) during the curation of disease maps.

#### Maps exploration via Web platforms

We also discussed how to explore and analyze the content of the disease maps. In this area, one of the first platforms for sharing disease maps as CellDesigner diagrams was Payao [16], followed by iPathways+ [4]. Their functionality was extended by tools like Molecular Interaction NEtwoRks VisuAlization (MINERVA) platform [6] and NaviCell [14], developed by the DMC members. They allow for visualization of large CellDesigner and SBGN diagrams using the Google Maps Application Programming Interface (API) to provide interactive annotation to maps’ elements and enable overlay of experimental data on top of these maps. Another solution for browsing large maps are various complexity management techniques such as expand–collapse and hide–show featured by the Newt pathway editor [8]. However, an open issue is the exploration and integration of simulation results from the associated models. A rough shortcut is currently available via visualization: e.g. the outcomes of flux balance analysis can be shown by different thickness and color of corresponding reactions on the map, as in Escher [21]. Another example, the iVUN system (interactive Visualization of Uncertain biochemical reaction Networks) [22], uses the kinetic parameters encoded in the map directly via the visualization interface to run simulations. Finally, the recently upgraded COnstraint-Based Reconstruction and Analysis (COBRA) Toolbox [20] introduces a built-in visualization functionality for constraint-based modeling results and enables visualization of modeling results via the MINERVA platform. Overall, current platforms for analysis and visualization are Web-based, and with the increasing size of disease maps, it is important to ensure scalability of expensive operations such as layout and simulation. The increase of client-side computing power allows to use local resources for some work and use the Web server for heavy computations like graph layout. A milestone in the direction of in-depth map exploration will be Web-based visualization of simulation results together with the contents of a disease map, or its parts, used for the simulation.

#### Integrating maps in a shared repository

Another challenge that requires proper tools is the integration of maps into a repository. As disease maps projects mature, it is natural to break up large complex maps into smaller modules, which can be used independently or composed into the full map. This asks for a platform to manage multiple maps simultaneously, and cross-link their content. Currently, MINERVA and NaviCell offer support in creating a single hierarchical multi-modular disease map. A challenge that remains to be addressed is a repository spanning multiple disease maps, allowing us to query resources of various disease domains, either by keyword or by network neighborhood. For this to happen, we need to propose solutions for versioning and comparing different maps, also taking into account different annotations and context of particular projects with the aim to converge into the common standard of disease maps annotations and representation. Often, the lossless conversion between formats like SBML, SBGN or Biological Pathway Exchange (BioPAX) [10–12] is not possible. Therefore, it is crucial to develop a framework for a unifying notation for encoding the disease mechanisms and annotating them (discussed in the section ‘Biocuration and knowledge representation standards’), supported by converters minimizing the information loss on translation. A good step in this direction may be a repository of uniform, reusable modules and models of pathways that are common for multiple disorders, and can be used across many projects (discussed in the section ‘Map complexity management’). Efforts like FAIRDOMhub, the NDex platform and the Physiome Model Repository go in a similar direction [23, 25, 27]. The effective use of a shared repository is only possible with a powerful set of queries including graph-based ones such as shortest paths between a specified set of molecules and common target of a gene set [28]. Here, a milestone will be a translation of one or more common modules between different disease maps. Another important goal to be reached is enabling communication between different disease maps, allowing to query their resources.

### Biocuration and knowledge representation standards

Biocuration of a disease map is a difficult task that heavily depends on the expertise of the curator. A clearly defined set of best practices can facilitate this process, similarly to protocols for construction of biomodels [29]. External resources like Gene2Disease or MalaCards, and tools like Integrated Network and Dynamical Reasoning Assembler (INDRA) [30–32] can help in organizing and referencing the disease-related knowledge integrated into a map.

#### Curation standards

A number of curation standards can help with harmonizing the content in various disease maps. Graphical notation and modeling languages like SBGN, SBML or CellML [11, 12, 26] offer good guidance in encoding molecular networks, while annotation of biological entities according to the Minimal Information Requested In the Annotation of biochemical Models (MIRIAM) guidelines is supported by the Identifiers.org infrastructure [33]. Whenever modeling-level description is available, the model structure can be automatically checked for consistency, e.g. to detect divergent reactions, or negative concentrations of molecules [34]. Continuous checks for correctness against these standards and resources are a key activity for developing useful disease maps. However, the specificity of certain disease mechanisms is often difficult to describe in a standardized manner. Encoding and annotating protein complexes or specific post-translational modifications in a diagram may be challenging for the curator, when the proper balance between clarity and precision is not obvious. Thus, it is important to establish a set of quality indicators for the curated mechanisms indicating their usefulness and the precision of the underlying information.

#### Map updates

The standards mentioned above describe the format of the content. Another important aspect that requires attention is the relevance to the disease area—keeping the content up-to-date and relevant for current and upcoming analytical challenges. This requires dedicated curation effort, but also a community of users in the field who evaluate the content and assess its relevance for the disease of interest. Thus, supporting a given disease map by accompanying social networking tools, like discussion forums, may help catalyze the communication. From the computational point of view, text mining solutions may be used to identify potentially relevant mechanisms to include or review. These suggestions can be in turn discussed openly with the community, encouraging discussion and engagement. Testing such a text mining-based update system and comparing it across different disease maps may provide new ideas how to accelerate the time-consuming curation process. Additionally, this may lead to improvement of the algorithms of text mining supporting the curation, as they are tested against manually curated information.

#### Knowledge representation consistency

The DMC projects cover various pathologies and are at different stages of development. This diversity results in varying depth of curation for particular diseases and their mechanisms. For instance, knowledge about specific mutations and their mechanisms is important for the cancer field, while chronic disorders may put less emphasis on it. For this reason, the content of different disease maps should be reused with care. Molecular pathways implemented in a map for neurodegenerative diseases may be relevant in inflammatory disorders, but they might have to be modified or extended. Therefore, consistent and precise annotation is necessary for both appropriate use and successful reuse of curated content. Although platforms like MINERVA offer an annotation consistency check, the verification takes place after the curated content is uploaded to the platform. A curation tool checking for annotation consistency on-the-fly would help to avoid errors and omissions, improving the quality of generated content and reducing the curator’s burden.

#### Connecting maps to disease hallmarks

Another challenge curators face is to design the map in such a way that end users can recognize the mechanisms of the disease and tell them apart from the normal, physiological function of a given pathway. Also, users often interpret the map based on their individual data sets, for instance for subgroups of patients, or specific cell lines.

While curating the map’s content, it is important to evaluate it methodically for the relevance to each disease area. Replication of hallmark findings in a given domain is often tangible, as many appropriate data sets are now publicly available, either via general repositories of molecular phenotypes, such as Gene Expression Omnibus [35] and the Expression Atlas [36] or disease-specific resources such as the Genomic Data Commons [37] and the Human Protein Atlas [38] in the case of cancer. Identification of differentially expressed molecules and their visualization on the map will help to refine the map’s content, but also will be a demonstration of its utility. A series of such analyses may help to calculate significance and vulnerability scores, describing how strongly a given mechanism is implicated in the disease, and how often it is perturbed. Benchmarking scenarios, describing these *in silico* validation experiments, are a necessary component of disease map development. Such scenarios and benchmark data sets will have to take into account the disease heterogeneity and differences in statistical approaches used for data preparation across studies.

### Map complexity management

Disease maps aim to describe disease mechanisms, which often span across multiple scales of human physiology and involve numerous cross-talking pathways. This comes with the challenge of meaningful organization of such complex knowledge. Thus, complexity management in our case aims to resolve the perception difficulty of different scales and mechanisms without losing the understanding of the disease as a whole.

Complexity management foundations for disease maps are distilling the relevant content to the disease context, highlighting the mechanisms critical for the pathology, categorizing the mechanisms based on their general biological relevance and creating high-level, abstract views of relationships between key concepts. These approaches are used already at the stage of curating the maps’ content.

#### Network complexity

Densely connected biological networks are impossible to draw without edge crossing (nonplanar graphs). A currently applied approach is to create multiple instances of (to clone) a molecule in various contexts (different compartments, pathways or modifications), which reduces visual clutter. This task can be automated by an algorithm suggesting when to clone a certain molecule to improve overall graph perception [39]. Similarly, clearly separable modules of a disease map can be transformed into submaps, linked hierarchically to the overview map. At the same time, visualization and management of such distributed content become more difficult, as different instances of the same molecule, or separate submaps, have to be meaningfully searched and explored. Development of tools for exploration of hierarchically abstracted and modularized networks is an important milestone on the road toward managing network complexity. Testing the existing functionality of Newt for collapsing subnetworks, especially for large-scale disease maps, will help to better specify challenges in front of such tools.

Finally, we noticed that in the field of electrical engineering, which was a source of inspiration for developing standards for graphical network representations, established conventions exist for representing crossing wires on the electrical diagrams. As creating network diagrams completely free of edge crossing does not seem to be possible or useful, developing standards on resolving possible misinterpretations would be a useful step in managing complexity of large disease maps.

#### Scale complexity

Another group of complexity management techniques concerns map visualization. These include semantic zooming into diagrams [6, 7], collapsing and expanding subnetworks in a diagram [8] or bundling edges to discover structure of dense networks [40]. One important type of semantic zooming subdivides different content types among multiple layers, where the zoom level defines the level of complexity seen by the user. For instance, the highest zoom level could show the most generic physiological view, e.g. the tissue or organ affected by the disease, the zoom layer below would show cell type relationships in the tissue, while subsequent zooms would show different levels of complexity of underlying cellular and molecular networks. Visualization of these complex networks at low granularity can be facilitated by representing network motifs (commonly encountered graph structures, like phosphorylation or complex formation) as recognizable symbols, or highlighting the most relevant molecules for the disease. This hierarchical way of layered display can be complemented by ‘vertical’ layers, showing separately different classes of molecular processes, e.g. transcription, signaling or metabolism.

#### Layout complexity

Hierarchical layers allow complexity management at the overview level for easier navigation to a particular area of the map. However, when examining details of molecular processes, users need tools to disentangle dense bundles of interactions and relate the content in front of their eyes to the rest of the disease map. Display of such local views can be implemented with the help of dynamic layouts, where the wiring of the diagram is temporarily changed in the area examined by the user to better reflect current context. Interactively changing the layout on-the-fly can be foreseen for the local views because of their small size. For instance, the technique of hyperbolic trees may allow us to remove local edge crossings in an area of the map, which would be infeasible for the entire map [41]. The local topology of the network can also be adapted to minimize the curvature of locally viewed edges [42], or it can be modified to reflect the uploaded data sets. In these data-driven layouts differentially regulated molecules can become larger and more central, while flux balance analysis results may change the length of the edges to reflect the reaction rate. There are alternative methods for creating data-driven layouts of biological networks, based on nonlinear dimension reduction constrained by the network structure [43]. These and other complex graph visualization methods such as hierarchical bundling of smoothed edges [44] can greatly facilitate understanding the complex structure of connections between the objects on the map and its relation to the studied data sets.

#### Managing technical complexity

A less conceptual but not less important aspect of managing complexity of disease maps is related to technical problems, i.e. it concerns a set of questions related to performance and interoperability.

Despite the development of a new generation of network editors, efficient manipulations needed for constructing and maintaining disease maps with thousands of nodes remain challenging. Here, one could explore the possibilities of existing approaches for complex and multiscale visualizations used in other domains such as the Web Graphics Library (WebGL). For instance, while dealing with large and complex networks, one can reuse existing methods of advanced memory caching that avoid keeping the whole complex network in memory, like it is done in Google Maps for smooth browsing of huge raster geographical images.

The interoperability between existing standards approved by the community, such as SBGN Markup Language (SBGN-ML), SBML 3.0 with Layout and Render extension and *de facto* standards used to construct most of disease maps, like the CellDesigner proprietary SBML extension, remains a challenge. However, this aspect happened to be relatively inexpensive to improve. For instance, at the time of writing, a new fully functional bidirectional converter from CellDesigner to SBGN-ML has been developed as a collaborative effort between DMC members (https://github.com/royludo/cd2sbgnml). Such tools will allow the use of the rich computational systems biology toolkit to analyze the existing collections of disease maps.

### Applications of disease maps

The way disease maps are used drives the curation of the content and indicates directions for technology development [45]. Disease maps are created for various purposes, for instance as a didactic resource, a knowledge repository, a platform to visualize data or a collection of predictive molecular signatures. These use cases reflect different stages of development of a disease map, when its contents are continuously refined from a collection of most known mechanisms of a given disease (‘hallmarks’) through verification against established expertise and available experimental data.

#### Access to bioinformatic databases

Disease maps applications that focus on knowledge exploration require easy and direct access to various data resources. MINERVA and NaviCell platforms provide such access to a number of annotation sources, like HUGO Gene Nomenclature Committee (HGNC), UniProt, Chemical Entities of Biological Interest (ChEBI), PubChem or Gene Ontology [46–49]. From our experience, users can better understand representations of particular disease mechanisms if they can cross-check descriptions of the included molecules. However, advanced data interfaces are needed, such as querying pathway databases for entire sequences of reactions from Reactome or WikiPathways [50, 51]. Newt implements such functionality for drawing interactions. A corresponding feature for visual exploration of disease maps remains to be implemented.

#### Tissue and disease specificity

Visual navigation through complex content will be greatly facilitated by introducing visual tags for cell or tissue types on the maps. Highlighting elements or interactions unique for certain physiological environments is needed for users to disentangle complex bundles of reactions, and to understand them. Semantic zoom functionalities, already implemented to a certain degree in disease maps platforms (discussed in the section ‘Map complexity management’), need to be extended. When zooming into complex networks, the content should be presented with gradually increasing number of details, based on the complexity of underlying physiology and on the density of explored molecular networks.

Individual disease maps represent contextualized pictures of various pathologies. Comparing disease maps’ contents will help to identify deregulation of mechanisms specific to a given disorder, as well as pathways implicated in a number of pathologies. Such comparisons become tangible thanks to pipelines for data cross-linking and visualization of complex networks. Combined with patient-specific data, such exploratory analysis in maps of overlapping pathologies, like cancer subtypes, may support personalized medicine by facilitating interpretation of patient-specific drug resistance.

#### Health and disease data interpretation pipelines

Clinical applications of disease maps [45, 52] are close to the role of a Clinical Decision Support System, with an emphasis on exploration and interpretation of medically relevant data. Big health data, collected in great amounts by health-care providers and pharmaceutical companies, need to be structured and interpreted through visualization. This is a scenario where disease maps may provide a valuable context to large data sets, allowing meaningful filtering and summary of otherwise indigestible numbers. Initial steps in creating big health data pipelines to disease maps have been taken [45], where a disease map is used to visualize gene expression based on patients’ demographic data.

In the end, disease maps may be a great support to knowledge-based drug discovery using patients’ data, but only after drug databases can be linked with the maps’ content and supported by dedicated analytical pipelines. For instance, disease maps may become a platform for network data-driven drug response prediction. This will require identification and assessment of disease-rewired pathways, network analysis to identify a desired intervention set (target interactions or elements in the network) and mapping this intervention set back to drug databases, looking for secondary use of existing medications (drug repositioning).

The final goal of a disease map development is to become mathematically interpretable and to support clinical decisions in a given domain. Importantly, the process of refining and exploring a disease map itself provides knowledge building, even without an immediate clinical application. Although the map is created to be quantified and analyzed with data to predict a clinically relevant outcome, its qualitative interpretation can have a great value in hypothesis generation and for guiding experimental design. This is an important note to take into account when managing expectations about applications of a disease map.

### Use of maps for mathematical modeling

Disease maps are currently used to organize knowledge and to visualize data. The ultimate goals are however the generation of testable hypotheses, the identification of actionable targets and the support of clinical decision making. To achieve this, executable mathematical models are required. Depending of the required level of resolution, qualitative models (e.g. logical or Boolean models), or quantitative models (e.g. ordinary differential equations, stochastic differential equations or Markov jump processes) can be used. Yet, the formulation of mathematical models requires more information than the use of maps for visualization, and this generates additional challenges to address.

#### Construction of executable mathematical models from disease maps

The formulation of executable mathematical models requires information on molecular species and their interactions. For the formulation of qualitative models, information about the mode of interaction between molecular species is required (e.g. activating or inhibiting). This information can be extracted from SBGN Activity Flow maps [53, 54]. However, most of the available disease maps use SBGN Process Descriptions or a combination of SBGN Process Descriptions and SBGN Activity Flow diagrams. This complicates an automatic construction of a logical model substantially. For the formulation of quantitative models, information about the properties of reactions is necessary, including stoichiometry and reaction kinetics [55]. While stoichiometry should be encoded in SBGN Process Descriptions, the kinetic rate laws are usually missing. The definition of rate laws requires additional information or assumptions, e.g., that a reaction follows the law of mass action kinetics. Some efforts have been launched to generate logic and numerical models from pathway maps [56]. For instance, the ongoing work on automated translation of SBGN and CellDesigner formats into logical models may help to bridge the quantitative and qualitative applications of disease maps. However, this remains a challenging task, providing results of mixed quality. To support the construction of executable mathematical models from disease maps, the first milestone would be the definition of a standard operating procedure (SOP), which informs biocurators about the minimal information, which has to be implemented in the disease maps. In this context, the use of SBML for the model formation and automatic checking of model consistency might be more appropriate. An important issue is therefore to ensure a proper link between molecular processes and the phenotype of interest.

#### Parameterization or executable mathematical models

Quantitative mathematical models usually possess unknown parameters, e.g. binding affinities and degradation rates. To ensure that the models are predictive, these parameters have to be estimated from experimental data. This requires comprehensive data sets as well as computational methods for statistical inference.

Data sets are available in the literature and in established databases, such as BRENDA [57] and SABIO-RK [58]. However, most literature-based data sets are unstructured and difficult to assess. Furthermore, the quality of experimental data varies heavily. A milestone for any disease map project aiming at quantitative models therefore is the establishment of a database of general and disease-specific data. The databases could be created together with the disease maps, and encode essential qualitative properties as well as quantitative data. The databases established for different projects should ideally follow common standards.

To estimate the unknown parameters from the available data, an efficient computational pipeline is required. As disease maps usually possess hundreds or even thousands of state variables and parameters, the resulting computational complexity might be challenging for established toolboxes such as COmplex PAthway SImulator (COPASI) [59], Data2Dynamics [60], Parameter EStimation TOolbox (PESTO) [61] or PottersWheel [62]. Moreover, such a large number of variables will require an automated procedure to check parameter identifiability. A milestone is the establishment of a scalable computational pipeline, which is applicable to the standardized models and databases established in the disease map projects. Such a pipeline could combine efficient objective function and gradient evaluation methods [63] with advanced parallel optimization schemes [64].

#### Personalization of models using data

A parameterized quantitative model can in principle be used for decision support in the clinic. To provide patient-specific predictions, the model needs to be personalized with patient-specific information. While this is a procedure fairly easy to do with small models, such as the ones used in pharmacokinetic/pharmacodynamic modeling, it is much less so in the case of large maps with a great number of molecular partners. In recent studies, exome and transcriptome sequencing data of cancer cell lines have been used to set cell line-specific translation rates [65, 66]. In a similar study, the mRNA expression was used to predict the survival of individual neuroblastoma patients [67]. While both approaches were successful in the respective applications, transcription rates and mRNA levels can change in response to treatment. For an analysis of the long-term response of patients, alternative strategies may be necessary. A milestone in this respect will be to develop different individualization approaches and then assess them in a range of applications. In addition, disease-related functional variants need to be implemented to benefit from comprehensive sequencing and genome-wide association studies (GWAS).

### Summary

**Figure 2:**
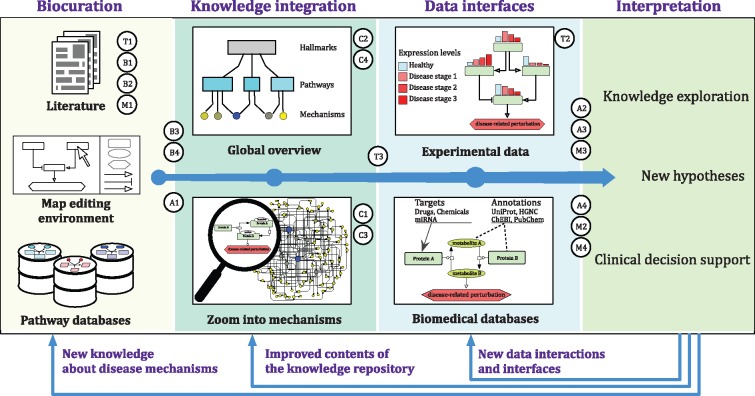
A life cycle of a disease map with the roadmap milestones. The figure illustrates the life cycle of a disease map, starting from the biocuration based on the relevant literature and available pathway databases. This knowledge is synthesized into a comprehensive repository: the disease map. Data interfaces and links to biomedical databases, together with accessible, visualized content allow for informed interpretation toward knowledge exploration, generation of new hypotheses or clinical decision support. The outcomes of the interpretation step link back to particular phases of the life cycle. ‘Data interfaces’ feedback describes the possibility of interconnecting additional data sources for better interpretation. ‘Synthesis’ feedback indicates improved knowledge organization within the disease map. ‘Biocuration’ feedback means introduction of new, validated hypothesis about the disease-related mechanisms. *Notes:* Milestones discussed for the Disease Maps Roadmap are mapped on the diagram as follows: T: Tools, T1: Modeling-oriented curation, T2: Visualization of simulation results, T3: Information exchange between maps; B: Biocuration standards, B1: Knowledge quality indicators, B2: Review of the text mining support, B3: On-the-fly consistency check, B4: Connecting mechanisms and disease hallmarks; C: Complexity management, C1: Dynamic subnetwork collapsing, C2: Algorithms for layered scale complexity, C3: Methods for dynamic layouts, C4: Handling large diagrams; A: Applications, A1: Cross-linking disease maps and pathway databases, A2: Data-based tissue specificity, A3: Data interpretation pipelines, A4: Quality assessment via *in silico* replication; M: Modeling, M1: Minimal information set for modeling, M2: Database of general and disease-specific data, M3: Scalable computational pipeline for models, M4: Model-based individualization approaches.

A ‘disease map lifecycle’, as shown in Figure 2, starts with curation and integration of knowledge about disease mechanisms. This collected knowledge, combined with experimental data and annotations from bioinformatics databases, supports better understanding of the disease and formulation of systems-level, data-driven hypotheses. The ‘disease map lifecycle’ is a dynamic process, as feedback from the interpretation of such contextualized knowledge leads to the design of further, tailored data interfaces, permits better consolidation of knowledge within the repository and may, if validated experimentally, introduce new knowledge about disease mechanisms for further curation and incorporation into the map. The milestones of the community-driven roadmap (Figure 1) are indicated in Figure 2.

#### Application example: drug repositioning

Signaling pathways implicated in human diseases create a complex network with redundant pathways. This complexity explains frequent failure of one-drug-one-target paradigm of treatment, resulting in drug resistance in patients. To overcome the robustness of the cellular signaling network, the treatment should be extended to a combination therapy scheme [68].

Disease maps allow integrating patient high-throughput data together with the information about biological metabolic and signaling machinery specific to a given disease. This in turn may help deciphering molecular patterns specific to each patient and finding the best combinations of candidates for therapeutic targeting. A simple drug repositioning scenario may involve creating data overlays for tissue-specific gene and protein expression and their visual analysis for spatial and temporal patterns in signaling cascades encoded in a given map. As disease maps platforms [6] provide a direct interface to DrugBank [69] and ChEMBL [70], the user can browse for drugs targeting the most interesting elements of the network directly via the visual interface. With a number of other such resources available, like STITCH [71], KEGG Drug [72], Cancer Therapeutics Response Portal [73], Kinome NetworkX [74] or NCGC pharmaceutical collection [75], this data interface can be extended to provide more extensive drug target search results.

Moreover, the digital and standardized form of disease maps enables their network structure to be easily extracted for high-throughput computational analysis, following the workflow established by the steps of visual exploration and analysis. The members of DMC performed such analyses to find synthetically interacting genes [76], predict drug synergy [77] or suggest complex intervention sets that open a possibility of drug repositioning [52, 78].

## Thematic highlight: mathematical modeling in human diseases research

The thematic highlight of the 2nd DMC meeting was mathematical modeling and disease maps. Building a computational model from a disease map is a process of transformation of a static literature-based representation into a dynamic executable format. This is important for a better understanding of how a disease progresses over time. It is also an environment where hypotheses and assumptions can be added and tested. Here, the prior knowledge (literature curation) can be integrated with newly generated data including omics data. Different types of computational models can be developed on the basis of the same pathway-based disease map. During the community meeting, we started reviewing and discussing possible approaches.

N. L. N. focused his presentation on the representation and modeling of allosteric proteins sensing calcium signals. Proteins with multiple binding sites, multiple independent features (such as binding partners, domains, conformations) and multi-subunit complexes are difficult to represent, let alone model. Trying to enumerate all molecular states leads to a combinatorial explosion of entities to model, and an even greater explosion of reactions to include. Some avenues allow to circumvent the problem, from rule-based modeling to abstract proteins representing probabilistic populations, or even implicit representations, e.g. Hill functions. Some of these approaches were illustrated by modeling Calmodulin, Calcineurin and CaMKII responses during synaptic plasticity.

J. H. presented parameter estimation methods based on adjoint sensitivities. These methods possess much better scalability properties than state-of-the-art approaches and facilitate the parameterization of large-scale models, potentially also executable models derived from disease maps. An application to a large-scale model of cancer signaling—essentially a disease map—was presented with more than a thousand chemical species and several thousands of unknown parameters [65]. J. H. demonstrated that the mechanistic model provides more accurate prediction for cell proliferation than statistical approaches.

R. M. T. F. discussed important differences between the notions of a reconstruction, a model and a map of molecular mechanisms in human physiology. He presented the Recon resource [79], the most complete reconstruction of human metabolism to date, and how in combination with constraint-based modeling it is used in systems-level biomedical research. The latest version of the reconstruction, called Recon3D [80], introduces structures of proteins and metabolites to the encoded reactions, and can be an important support to the canonical metabolic pathways in various disease maps. As an example, he discussed a map of mitochondrial metabolism, developed on the basis of Recon3D, that can support Parkinson's disease research.

A. Z. challenged the possibility of immediate use of disease maps in mathematical modeling, suggesting that they are currently playing a role of interactive encyclopedias rather than blueprints for chemical kinetics-based modeling of large reaction networks (structural network models). He argued that the disease maps rather reflect our knowledge in the corresponding domains together with its incompleteness and controversy. Thus, A. Z. coined a notion of executable encyclopedia as opposite to structural model, as a hypothetical approach based on pragmatic middle-out mathematical modeling as opposite to the pure bottom-up approach.

###

**Key Points**

- The Disease Maps Project is an interdisciplinary effort toward a systematic use of knowledge and data in research on human diseases.
- The proposed testable milestones will help Disease Maps' users, curators and technology developers to harmonize efforts and best practices.
- The suggested ‘lifecycle’ of a typical disease map project encompasses approaches available in the community and demonstrates applications.
- Mathematical modeling is discussed as an important aspect of Disease Maps, helping to refine their content and allowing to formulate predictions about disease mechanisms.

## Outcomes and outlook

The 2nd DMC meeting brought together curators of disease maps, developers of methodologies and tools and users. This allowed us to clarify objectives and use cases, and aligned them into a multi-lane roadmap for disease maps. The DMC will progress in parallel on several different lanes: tools, applications, curation standards, complexity management and mathematical modeling, at different paces, but in the same direction and with the same goal. Importantly, there are stages of the roadmap where the milestones align across the lanes. These will be treated with priority by the community.

Our discussions brought up a number of resources that we, disease maps curators and users, can benefit from. Participation of leaders of the Physiome and Recon projects [79, 81] led to ideas on how to capitalize on existing and well-structured knowledge and methods they developed. We reviewed current and upcoming interfaces to pathway databases and data analysis pipelines that will help us to curate and interpret the maps’ content.

This productive series of meetings will continue. The 3rd DMC meeting is scheduled for June 2018 in Paris, hosted by Institut Curie (http://disease-maps.org/events). We aim to review and update the roadmap and enlarge the community. Most importantly, we would like to maintain the atmosphere of collaboration and open exchange within the community, which is the key to improvement and further development of the Disease Maps Project. There are several tools, approaches and platforms developed by DMC members. Exposure of the participants to these resources will allow active exchange of know-how, and parallel hands-on tutorials will be provided.

## Notes

**Marek Ostaszewski** is a scientist and a project manager at the Luxembourg Centre for Systems Biomedicine (LCSB), working on IT applied to knowledge management in systems biomedicine, in particular in Parkinson's disease, including clinical research.

## Notes

**Stephan Gebel** is a molecular biologist, focusing on disease-related molecular pathways. He works at the Luxembourg Centre for Systems Biomedicine (LCSB) as the project manager of the Parkinson’s Disease map, coordinating content curation and outreach to map's users.

## Notes

**Inna Kuperstein** is a researcher at Institut Curie, Paris, France, she is a coordinator of the Atlas of Cancer Signalling Networks (ACSN) project for development and analysis of detailed disease maps, development of tools and modeling maps for predicting drug response.

## Notes

**Alexander Mazein** is a senior researcher at the European Institute for Systems Biology and Medicine, focused on a comprehensive representation of disease mechanisms and data interpretation in translational medicine projects.

## Notes

**Andrei Zinovyev** is the scientific coordinator of the Computational Systems Biology of Cancer team at Institut Curie, Paris, France. His research focuses on high-throughput biological data analysis, complexity reduction, and modeling of biological networks involved in tumorigenesis and tumoral progression.

## Notes

**Ugur Dogrusoz** is the head of i-Vis Lab and a professor of Computer Engineering in Bilkent University. He is currently an SBGN editor and works on network visualization methods and tools.

## Notes

**Jan Hasenauer** leads a research group at the Institute of Computational Biology of the Helmholtz Zentrum München, focused on the development and application of methods for the data-driven modeling of biological processes.

## Notes

**Ronan M. T. Fleming** leads an interdisciplinary research group of mathematical, computational and experimental biologists at the Leiden Academic Centre for Drug Research. Their interest is to increase the predictive fidelity of biomolecular network models for characterization and explanation of human diseases.

## Notes

**Nicolas Le Novère** coordinated the development of BioModels and was a major figure behind the development of a coordinated set of standards in systems biology, including SBML, SBGN and the MIRIAM guidelines.

## Notes

**Piotr Gawron** is a researcher at the Luxembourg Centre for Systems Biomedicine (LCSB) developing tools for visualization and exploration of complex molecular networks.

## Notes

**Thomas Ligon** is a guest scientist at the Faculty of Physics and Center for NanoScience (CeNS), Ludwig-Maximilians-Universität in Munich. His work includes computational simulation and parameter estimation of biological models.

## Notes

**Anna Niarakis** is an associate professor at Université d’Évry-Val-d'Essonne. Her research focuses on application of computational systems biology approaches in human diseases, including the construction of disease maps, network integration and dynamical modeling.

## Notes

**David Nickerson** is an Aotearoa Fellow at the Auckland Bioengineering Institute, working on a variety of computational physiology projects. He is also involved in several computational modeling standardization communities.

## Notes

**Daniel Weindl** is a postdoctoral researcher at the Helmholtz Zentrum München (Hasenauer Lab), working on parameter inference of large-scale dynamic models of signaling pathways.

## Notes

**Rudi Balling** is the director of the Luxembourg Centre for Systems Biomedicine (LCSB). His main interest is interdisciplinary research in human diseases realized by combining expertise in mathematics, computational biology and clinical research.

## Notes

**Emmanuel Barillot** is the head of the Cancer and Genome: Bioinformatics, Biostatistics and Epidemiology of a Complex System department and scientific director of the bioinformatics platform at Institut Curie, Paris, France. His research focuses on methodological development and statistical analysis of high-throughput biological data and modeling with the aim to improve therapeutic treatments of cancer.

## Notes

**Charles Auffray** is the President and Founding Director of the European Institute for Systems Biology and Medicine. He develops a systems approach to complex diseases, integrating functional genomics, mathematical, physical and computational concepts and tools.

## Notes

**Reinhard Schneider** is the Head of the Bioinformatics Core facility at the Luxembourg Centre for Systems Biomedicine (LCSB). His team develops solutions for efficient data integration, interpretation and exchange between the experimental, theoretical and medical domains.
